# How Stress Influences the Dynamic Plasticity of the Brain’s Extracellular Matrix

**DOI:** 10.3389/fncel.2021.814287

**Published:** 2022-01-25

**Authors:** Blake J. Laham, Elizabeth Gould

**Affiliations:** Princeton Neuroscience Institute, Princeton University, Princeton, NJ, United States

**Keywords:** perineuronal net, extracellular matrix, stress, hippocampus, prefrontal cortex, amygdala

## Abstract

Diffuse and structured extracellular matrix (ECM) comprise ∼20% of the brain’s volume and play important roles in development and adult plasticity. Perineuronal nets (PNNs), specialized ECM structures that surround certain types of neurons in the brain, emerge during the postnatal period, making their development and maintenance potentially sensitive to experience. Recent studies have shown that stress affects diffuse ECM as well as PNNs, and that such effects are dependent on life stage and brain region. Given that the ECM participates in synaptic plasticity, the generation of neuronal oscillations, and synchronous firing across brain regions, all of which have been linked to cognition and emotional regulation, ECM components may be candidate therapeutic targets for stress-induced neuropsychiatric disease. This review considers the influence of stress over diffuse and structured ECM during postnatal life with a focus on functional outcomes and the potential for translational relevance.

## Introduction

In recent decades, it has been recognized that a comprehensive view of brain function requires considering the entirety of the brain’s microenvironment, including neurons, non-neuronal cells, the vasculature, and the extracellular matrix (ECM). Compelling evidence now suggests that each of these entities contributes not just to the brain’s structure, but to its function and its ability to respond to experience with adaptive changes (Song and Dityatev, 2018; Cope and Gould, 2019). Perhaps because of their malleability, each of these constituents has also been linked to neuropsychiatric disease. A growing literature suggests that the ECM is particularly important for a wide range of processes that are critical components of experience-dependent change in brain function, including synaptic plasticity (De Luca et al., 2020), neuronal oscillations (Carceller et al., 2020; Wingert and Sorg, 2021), and network connectivity (Bucher et al., 2021; Christensen et al., 2021). Since plasticity at the synapse, neuronal synchrony within a brain region, and coherence across brain regions are critical for healthy brain function, understanding ECM involvement in these processes may also elucidate its role in brain pathology.

The ECM includes two main categories: diffuse and structured (Nicholson and Syková, 1998; Krishnaswamy et al., 2019). Diffuse ECM fills the spaces among neurons, glia, and the brain’s microvasculature, and consists of polysaccharides, proteins, glycoproteins, and glycosaminoglycans. Although the ECM was initially thought to function primarily as a supportive substrate, essentially holding brain cells together (Celio, 1999; Cope and Gould, 2019), it is now known to play major roles in both the developing and adult brain, including the guidance of migrating neurons and growing axons, attracting and repelling astrocytes and microglia, and regulating neurotransmitter receptor availability at the synapse (Franco and Müller, 2011; Lubbers et al., 2014). Structured ECM has a similar general chemical composition to diffuse ECM, but due to a high concentration of chondroitin sulfate proteoglycans (CSPGs), it forms a lattice-like assembly around certain types of neurons, primarily inhibitory interneurons. These structures, known as perineuronal nets (PNNs), typically surround the cell bodies and proximal dendrites of neurons throughout the brain. Multiple functions have been ascribed to PNNs, including to protect neurons from free radical damage, to limit the formation of unnecessary synapses, and to functionally stabilize neuronal systems by increasing inhibitory tone after development has ended (Sorg et al., 2016; Bucher et al., 2021; Burket et al., 2021). Perhaps not surprisingly given the number of important neural processes linked to diffuse and structured ECM, studies suggest that both types are associated with key brain functions, including learning and memory, as well as emotional processing.

Stress is known to affect cognitive processes, as well as mood regulation (McEwen, 2010), raising questions about whether it does so, at least in part, by impacting the ECM. Indeed, a growing literature indicates that stress impacts the ECM both during development and in adulthood (Tables 1, 2). This mini-review first provides a general overview of studies linking the ECM to brain function during development and adulthood, and then focuses on how stress may influence both diffuse and structured ECM, including discussion about potential functional consequences.

**TABLE 1:** Stress effects on diffuse ECM components.

Stressor and timing	Species/strain/ sex	Brain region	ECM component effect*	References
Maternal separation P2-14 (3 h daily)	Rat/Wistar/male	HIP	Reelin decrease during development, increase in adulthood	Zhang et al., 2013
Maternal separation P1- 14 (3 h daily)	Rat/SD/male	HIP	Reelin increase in adulthood	Wang et al., 2018
Corticosterone injection in adulthood	Rat/SD/male	HIP, PFC	Reelin decrease in adulthood	Lebedeva et al., 2020
Chronic unpredictable mild stress for 6 weeks in adulthood	Mice/apoE/male	HIP, PFC	Reelin decrease in adulthood	Zhang et al., 2021
Chronic unpredictable stress for 6 weeks in adulthood	Rat/SD/male	HIP, FC	Laminin decrease	Laifenfeld et al., 2005a
Social defeat during adolescence	Mice/C57/male	HIP, NAc	Laminin decrease	Rodríguez-Arias et al., 2017

*Compared to unstressed controls.

*SD, Sprague Dawley; apoE, apolipoprotein E; FC, frontal cortex; HIP, hippocampus; PFC, prefrontal cortex; NAc, nucleus accumbens.*

**TABLE 2:** Stress effects on PNNs.

Stressor and timing	Species/strain/ sex	Brain region	PNN effect*	References
Scarcity/adversity P8-P12	Rat/SD/male	BLA	Lower PNN intensity at P23	Santiago et al., 2018
Scarcity/adversity P4-P10	Rat/SD/male, female	BLA	Higher PNN number in right male BLA at P28	Guadagno et al., 2020
Maternal separation P2-P20 (4 h daily)	Rat/SD/male, female	BLA, PFC	Lower PFC PNN number at P20; higher PNN intensity in adult male PFC, adult female BLA	Gildawie et al., 2020
Maternal separation P2-P14 (3 h daily)	Rat/SD/male, female	BLA, PFC	No change in PNN number in BLA or PFC at P18 or P28	Richardson et al., 2021
Maternal separation P2-P14 (3 h daily)	Rat/SD/male	HIP	Higher PNN CSPGs in adults	Dimatelis et al., 2013
Maternal separation and early weaning P2-P16 (4–8 h daily)	Mice/C57/male	HIP	Higher PNN intensity in adults	Murthy et al., 2019
Maternal separation P2-P20, social isolation P21-P35	Rat/SD/male, female	PFC	Lower PNN PV number, PNN intensity in females	Gildawie et al., 2021
Social isolation P21-P56	Mice/C57/male	HIP, PFC	Lower PNN number PFC	Ueno et al., 2017
Unpredictable chronic mild stress P28–42	Mice/C57/male, female	PFC	No change in PNN number	Page and Coutellier, 2018
Social isolation P21-P90	Mice/FVB/male	HIP, RC	Lower PNN number in HIP, RC	Klimczak et al., 2021
Social defeat (daily for 5 days) in adults, social isolation (3–60 days)	Rat/LE/male	HIP	Lower PNN CSPGs at 3 days, higher at 60 days post-stress	Koskinen et al., 2020
Restraint (6 h/day for 10 days) in adults	Rat/SD/male	HIP, BLA, HAB, RT, PFC	Higher PNN number in PFC; lower in HIP; increased intensity in RT, HAB	Pesarico et al., 2019
Chronic mild stress (10, 20, 30 days) in adults	Rat/SD/male	PFC	Lower PNN number at 20 days	Yu et al., 2020
Social defeat (daily for 5 days) in adults; social isolation (30–60 days)	Rat/Wistar/male	HIP	Higher PNN number	Riga et al., 2017

*Compared to unstressed controls.

*SD, Sprague Dawley; FVB, Friend Leukemia Virus, strain B; LE, Long Evans; PV, parvalbumin; PNN, perineuronal net; BLA, basolateral amygdala; HIP, hippocampus; RC, retrosplenial cortex; HAB, habenula; RT, reticular thalamic nucleus; PFC, prefrontal cortex.*

## Extracellular Matrix Function During Development and in Adulthood

Numerous studies have shown that diffuse ECM plays both subtle and vital roles in brain development. With regard to the latter, the glycoprotein laminin is critical for neural tube closure, making its knockout lethal (Miner et al., 1998). After the basic structure of the brain is formed, other ECM molecules, such as the glycoprotein reelin and the glycosaminoglycan hyaluronan, help to coordinate neuronal migration, axon guidance, and synaptogenesis (Borrell et al., 2007; Honda et al., 2011; Vaswani and Blaess, 2016). ECM molecules also sequester growth factors, chemokines, and additional molecules with attractant and repellent properties, all of which work to coordinate brain development. Diffuse CSPGs also play a role in multiple cellular events during development (Sirko et al., 2007; Zimmer et al., 2010), and their sulfation patterns are known to influence their involvement in events such as neuronal migration and maturation (Maeda et al., 2010, 2011).

As development proceeds, molecules of the diffuse ECM, such as reelin, undergo changes in abundance and function. In adulthood, reelin takes on a new function of enhancing synaptic plasticity at excitatory synapses. Reelin facilitates this function *via* its binding to the apolipoprotein E (apoE) receptor 2, which forms a complex with NMDA receptors (Beffert et al., 2005; Korwek et al., 2009). Reelin also functions in stimulating adult neurogenesis and dendritic spine formation in the hippocampus (Pujadas et al., 2010; Sibbe et al., 2015). Hyaluronan seems to have the opposite effect of reelin on adult neurogenesis in that its binding to the CD44 receptor reduces the production of new neurons in the hippocampus. Hyaluronan increases in the aging brain and may play a causal role in age-related reductions in adult neurogenesis (Su et al., 2017). Hyaluronan also participates in synaptic plasticity by regulating dendritic calcium channels (Kochlamazashvili et al., 2010).

During the postnatal period, the ECM surrounding a subset of neurons condense and forms PNNs, with CSPGs as major components. The lattice-like shape PNNs comes from the organization of CSPGs that bind the base of PNNs, which is hyaluronan, also a component of the diffuse ECM. The main CSPGs in the brain include aggrecan, neurocan, brevican, versican, and phosphocan, and their expression amounts are region-specific (Dauth et al., 2016; Pantazopoulos et al., 2021). Although speculative, the heterogeneous combination of PNN components may provide specialized function (Dauth et al., 2016). In addition, PNNs may have different functions depending on the sulfation patterns of their CSPGs, with some patterns conferring greater plasticity than others (Miyata et al., 2012; Yang et al., 2017).

The developmental appearance of PNNs in some brain regions coincides with the closure of critical periods, including the emergence of binocular vision in the visual cortex (Hensch and Quinlan, 2018) and leptin sensitivity in the hypothalamus (Mirzadeh et al., 2019). In adulthood, PNNs are thought to restrict plasticity, both by preventing the ingrowth of axons and the mobilization of neurotransmitter receptors at the synapse. In some cases, PNNs have been associated with increased firing of neurons they surround, which are primarily parvalbumin+ inhibitory interneurons in the neocortex and hippocampus (Sorg et al., 2016; Wingert and Sorg, 2021). The functional consequences of PNNs seem to differ depending on brain region, cell type, and behavioral task, with some studies showing that PNNs facilitate learning and memory, while others show they have an inhibitory effect on these processes (Paylor et al., 2018; Anderson et al., 2020; Carulli et al., 2020; Cope et al., 2021; Wingert and Sorg, 2021). The overall picture that is emerging is one that is common in biology—an inverted U-shaped curve exists where atypically low or high PNNs impair function. In addition to effects on behavior, PNNs have been shown to regulate neural correlates of cognitive function, including synaptic plasticity, neuronal oscillations, and neuronal synchrony across brain regions (Sorg et al., 2016; Bucher et al., 2021; Wingert and Sorg, 2021). These electrophysiological phenomena are also critical for behaviors associated with emotional regulation, and have been shown to be stress-sensitive (Murthy and Gould, 2020; Tomar et al., 2021), raising the possibility that stress-induced changes in brain function and behavior might occur through changes in the ECM. A growing body of evidence suggests that stress impacts both diffuse and structured ECM during development and in adulthood. The majority of studies investigating the effects of stress on the ECM have focused on stress-susceptible brain regions, including the hippocampus, the prefrontal cortex, and the amygdala, which play crucial roles in cognitive function and emotional processing (McEwen et al., 2015; Smith and Pollak, 2020).

## Stress Effects on Diffuse Extracellular Matrix

Developmental stress exhibits different effects on reelin signaling depending on the age at which the brains are investigated (Table 1). Postnatal stress has been shown to first reduce reelin expression in the hippocampus, and then show a compensatory rebound and overshoot as animals reach adulthood (Zhang et al., 2013). The postnatal stress-induced increase in adult hippocampal reelin expression can be augmented by exposure to a stressful learning paradigm, such as contextual fear conditioning. This effect is accompanied by enhanced hippocampal LTP and dendritic spine density (Wang et al., 2018). These findings suggest that postnatal stress-induced latent increases in reelin expression may serve an adaptive function. In contrast, chronic stress or chronic glucocorticoid treatment in adulthood decrease reelin expression in the hippocampus and prefrontal cortex (Lebedeva et al., 2020; Zhang et al., 2021). Studies additionally suggest that reelin signaling through the apoE receptor is important for mitigating stress-induced behavioral dysfunction, especially in older mice (Zhang et al., 2021). These findings suggest that developmental and acute adult stress may produce adaptive stress responses through augmented reelin signaling, while chronic adult stress may lead to dysfunction *via* a reduction in reelin expression. Along these lines, it may be relevant that antidepressant drug treatment reverses stress-induced decreases in reelin expression (Fenton et al., 2015; Johnston et al., 2020), and that hippocampal reelin infusions can reverse stress-induced behavioral dysfunction (Brymer et al., 2020).

Laminin expression is also decreased after chronic stress in both the adult hippocampus and frontal cortex (Laifenfeld et al., 2005a; Rodríguez-Arias et al., 2017). Similar to what has been observed with reelin, the stress-induced decrease in laminin can be reversed with antidepressant treatment (Laifenfeld et al., 2005a). Hyaluronan signaling also seems to play a protective role in mediating stress effects, as mice lacking the hyaluronan receptor CD44 exhibit exacerbated stress-induced behavioral dysfunction, as well as reduced brain levels of the neuromodulators serotonin and dopamine (Barzilay et al., 2016). Collectively, the overall picture suggests that components of diffuse ECM are stress-sensitive and potentially involved in adaptive mechanisms enhancing the ability to appropriately respond to stress and buffer against stress-induced pathology.

## Stress Effects on Perineuronal Nets

Several studies have investigated the effects of postnatal and adult stress on PNNs (Table 2). These studies have produced mixed results, likely due to differences in the developmental stage of stress exposure, the type of stressor, as well as the duration of time between stress and brain examination. An additional reason for potential discrepancies may be due to measures used to assess PNNs, which most commonly rely on the binding of an exogenous fluorophore-conjugated plant lectin Wisteria floribunda agglutinin (WFA). Using this approach, researchers most often quantify numbers of WFA+ cells or the intensity of WFA+ cells to assess whether PNNs have changed. WFA is not an endogenous component of PNNs, and although its binding site is known (Nadanaka et al., 2020), it does not label all PNNs (Yamada and Jinno, 2017; Ueno et al., 2018). Thus, changes in WFA labeling may be open to multiple interpretations. One study showed that postnatal stress using a scarcity/adversity model led to reduced PNN intensity in a subregion of the basolateral amygdala (Santiago et al., 2018), while another study using the same model reported increased PNN cell numbers but only on the right side of the amygdala in males (Guadagno et al., 2020). Two additional studies using maternal separation have reported either increased PNN intensity, but only in females (Gildawie et al., 2020), or no differences in PNN measures (Richardson et al., 2021). Although all of these studies used a similar method for identifying PNNs, they did not all use the same measure (WFA+ cell number vs. WFA intensity), and none used a label of a specific CSPG, such as aggrecan. It may be relevant that these studies also examined different time points after postnatal stress (Table 2), making it difficult to determine whether the effects would be more similar if the same time point had been examined.

Available evidence suggests a complex set of results from stress studies in the hippocampus and prefrontal cortex, with some studies showing decreased number or intensity of PNNs, others an increase, and others reporting no effects at all (Table 2). Examining the papers as a group, however, suggests that stress may have variable effects on PNNs depending on the duration of time after stress exposure. Several studies show decreased PNNs immediately after stress (Ueno et al., 2017; Pesarico et al., 2019; Gildawie et al., 2020, 2021; Koskinen et al., 2020; Yu et al., 2020; Klimczak et al., 2021), and a rebound increase in PNNs as time passes following stress cessation (Dimatelis et al., 2013; Riga et al., 2017; Murthy et al., 2019; Koskinen et al., 2020; Gildawie et al., 2021). Although some findings do not fit with this summary (Page and Coutellier, 2018; Richardson et al., 2021), the majority of studies suggest a stress-induced trajectory involving first suppression followed by an overshooting rebound, similar to what has been observed for reelin signaling (Zhang et al., 2013). This is in line with other theories of stress effects on the ECM, particularly the idea that depressive-like symptoms emerge after an “incubation period,” which involves latent increases in PNNs and accompanying plasticity reduction (Koskinen et al., 2020; Spijker et al., 2020). It should be recognized that outside of critical periods, PNNs are capable of rapid remodeling, as has been recently demonstrated in several studies (Marchand and Schwartz, 2020; Pantazopoulos et al., 2020; Uriarte et al., 2020). Evidence suggests that changes in PNNs can occur through alterations in neuronal activity both during development and in adulthood (Dityatev et al., 2007; Carstens et al., 2021; Devienne et al., 2021). Thus, stress-induced changes in neuronal activity (Della Valle et al., 2019; Murthy et al., 2019; Del Arco et al., 2020; Fee et al., 2020) could be a mechanism by which alterations in PNNs occur.

The extent to which stress-induced changes in PNNs represent adaptive or dysfunctional effects remains uncertain. Several studies show that reduced or increased PNN measures in the hippocampus, prefrontal cortex, and amygdala are associated with behavioral changes that are thought to reflect increased avoidance/threat and reduced stress coping behavior (Santiago et al., 2018; Murthy et al., 2019; Koskinen et al., 2020; Yu et al., 2020), as well as impaired cognitive function (Riga et al., 2017; Koskinen et al., 2020). Few studies have addressed causal relationships by including experimental manipulations of PNNs that restore healthy function after stress (Riga et al., 2017). Additional studies in non-stressed rodents have shown that reducing PNNs either by genetic manipulations or by enzymatic degradation can alter stress-susceptible behaviors in rodents, including avoidance, stress coping, cognitive function and substance use. For example, genetic deletion of neuronal membrane linking protein ankyrin-R or the transcription factor OTX2 reduces PNN expression and decreases avoidance of the open arms in an elevated plus maze task (Stevens et al., 2021; Vincent et al., 2021). Furthermore, degradation of PNNs using chondroitinase ABC facilitates extinction of drug-seeking behavior (Xue et al., 2014) and prevents both fear conditioning (Hylin et al., 2013) and cocaine-induced place preference (Slaker et al., 2015). However, studies have also shown that diminished PNNs can produce effects that mimic those of chronic stress, including increased threat responses (Santiago et al., 2018) and diminished cognitive function (Paylor et al., 2018). Since atypical behavioral states have been associated with both reduced or increased PNN measures in several brain regions, it seems likely that an optimal level of PNNs within a brain region may exist, which when disrupted produces behavioral dysfunction. Along these lines, it has been shown that antidepressant action on behavior and neuronal oscillations both require the presence of PNNs in the hippocampus (Donegan and Lodge, 2017), as well as the degradation of PNNs through the antidepressant-induced release of proteolytic enzymes by microglia (Alaiyed et al., 2019, 2020). Clearly, additional research is needed to better understand links among stress, PNNs, and behavior.

## Bridging the Gap Between Stress-Induced Changes in Extracellular Matrix and Behavioral Outcomes

Despite the variability of outcomes regarding stress effects on diffuse and structured ECM, both forms are sensitive to stress and their changes have been linked to alterations in behaviors associated with cognition and emotional processing. These findings raise questions about the mechanisms by which stress-induced ECM remodeling give rise to behavioral change. It is likely that the larger literature on the role of the ECM in electrophysiological function of relevant brain regions may provide clues. As mentioned earlier, both diffuse ECM and PNNs regulate synaptic plasticity (Sorg et al., 2016; Jakob et al., 2017), which has clear links to cognitive function (Dringenberg, 2020), raising a scenario whereby stress produces cognitive dysfunction by disrupting ECM components associated with optimal synaptic plasticity. ECM has also been linked to neuronal oscillations in both the gamma and theta frequency ranges (Murthy and Gould, 2020), which are important for both cognitive function (Mably and Colgin, 2018; Zielinski et al., 2020) and behaviors associated with emotional processing, including avoidance behavior (Padilla-Coreano et al., 2019). These results suggest that stress may produce cognitive dysfunction and enhance avoidance behavior through ECM-induced changes in neuronal oscillations. The ECM has also been linked to synchrony in rhythmic signaling across brain regions (Bucher et al., 2021), which has been shown to play crucial roles in healthy brain function in terms of learning and memory, as well as stress responsivity (Adhikari et al., 2010; Del Arco et al., 2020). It is conceivable that stress-induced ECM changes in these electrophysiological properties are responsible for stress-induced behavioral outcomes. Since antidepressant treatment restores stress-induced ECM changes and behavioral dysfunction in rodents, it seems plausible that this occurs through reversal of atypical synaptic plasticity, neuronal oscillations and/or circuit-level coherence. Antidepressant treatment has been shown to influence all of these electrophysiological measures (Law et al., 2016; Alaiyed et al., 2019; Logue et al., 2021).

## Future Studies

Future studies should directly investigate whether stress-induced changes in ECM lead to alterations in behavior through influences on electrophysiological properties at the synapse, among populations of neurons within a brain region, and across brain regions in the broader circuitry. Although these studies are likely to be informative, they will not provide a complete picture without considering other aspects of the brain’s microenvironment, such as glia, which are also known to be stress-responsive (Kaul et al., 2021) and participate in ECM remodeling (Strackeljan et al., 2021). Some studies have begun to test the involvement of microglia in regulating ECM in the context of both cognitive function (Alaiyed et al., 2019; Nguyen et al., 2020; Venturino et al., 2021) and emotional processing (Alaiyed et al., 2019; Venturino et al., 2021). Expanding these approaches to other brain regions, as well as other stress paradigms, should be illuminating. Lastly, finding ways to connect the experimental animal literature to humans will be important. Along these lines, it is relevant to note that the components of diffuse and structured ECM described in the rodent brain exist in the human brain (Fatemi et al., 2000; Laifenfeld et al., 2005b; Mauney et al., 2013). Furthermore, major depressive disorder and bipolar disorder, conditions linked to stress, have been associated with altered levels of diffuse ECM molecules, such as reelin, hyaluronan, and laminin (Fatemi et al., 2000; Guidotti et al., 2000; Laifenfeld et al., 2005b; Lubbers et al., 2014; Ventorp et al., 2016), and increased PNNs have been reported in the prefrontal cortex of suicide victims previously exposed to childhood maltreatment (Tanti et al., 2020). The similarities between rodent and human studies increase confidence that a better understanding of the connections between the ECM and stress-induced behavioral dysfunction in rodents may provide a window into stress-induced neuropsychiatric disease in humans.

## Author Contributions

BL and EG wrote and edited the manuscript. Both authors contributed to the article and approved the submitted version.

## Conflict of Interest

The authors declare that the research was conducted in the absence of any commercial or financial relationships that could be construed as a potential conflict of interest.

## Publisher’s Note

All claims expressed in this article are solely those of the authors and do not necessarily represent those of their affiliated organizations, or those of the publisher, the editors and the reviewers. Any product that may be evaluated in this article, or claim that may be made by its manufacturer, is not guaranteed or endorsed by the publisher.
